# Quantum ensembling methods for healthcare and life science

**DOI:** 10.1093/bib/bbag280

**Published:** 2026-06-04

**Authors:** Kahn Rhrissorrakrai, Kathleen E Hamilton, Prerana Bangalore Parthasarathy, Aldo Guzmán-Sáenz, Shreya Gupta, Tyler Alban, Filippo Utro, Laxmi Parida

**Affiliations:** IBM Research, Yorktown Heights, NY 10598, United States; Computational Science and Engineering Division, Oak Ridge National Laboratory, Oak Ridge, TN 37830, United States; Lerner Research Institute, Cleveland Clinic, Cleveland, OH44195, United States; IBM Research, Yorktown Heights, NY 10598, United States; Lerner Research Institute, Cleveland Clinic, Cleveland, OH44195, United States; Lerner Research Institute, Cleveland Clinic, Cleveland, OH44195, United States; IBM Research, Yorktown Heights, NY 10598, United States; IBM Research, Yorktown Heights, NY 10598, United States

**Keywords:** quantum machine learning, ensemble learning, renal cell carcinoma

## Abstract

Learning on sample-limited data is a challenge frequently encountered in many real-world applications. In this work we study how effective quantum ensemble models are when trained on a sample-limited data problem in healthcare and life sciences. We constructed multiple types of quantum ensembles for binary classification using up to 26 qubits in simulation and 56 qubits on quantum hardware. The ensembles include both variational and non-variational methods as well as introducing a new quantum ensemble cosine classifier with randomly sampled unitaries. Our ensemble designs use minimal trainable parameters but require long-range connections between qubits. We tested these quantum ensembles on synthetic datasets and gene expression data from renal cell carcinoma (RCC) patients with the task of predicting patient response to immunotherapy. From the performance observed in simulation and quantum hardware experiments using up to 56 qubits, we demonstrate how quantum embedding structure affects performance and discuss how to extract informative features and build models that can learn and generalize effectively. We also find that quantum ensemble cosine classifiers were effective in learning from few training data, and all quantum ensembles performed comparatively to classical ensembles while using significantly fewer learners. We confirmed this performance characteristic in a separate RCC validation cohort. We present these exploratory results in order to assist other researchers in the design of effective learning using ensembles, particularly for similarly size constrained problems. Incorporating quantum computing in these data constrained problems offers hope for a wide range of studies in healthcare and life sciences where biological samples are relatively scarce given the feature space to be explored.

## Introduction

Artificial intelligence and machine learning (AI/ML) have led to incredible advancements in healthcare and life science (HCLS) [1]. However, biological and healthcare data still pose many challenges for AI algorithms, including complexity and scale challenges [2], learning in sample-limited scenarios, model overfitting, saturated learning [3, 4], quadratic time and space complexity, and poor generalization. Concurrently, there has been meaningful progress in quantum computing, which may offer solutions to some of those limitations, such as improved generalization in training data limited contexts [5], and for specific use cases by tackling problems intractable for classical computing as well as providing possible, orthogonal interpretations to the vast complexity of biological systems [6, 7]. Quantum computing offers new strategies for accelerating drug discovery, aiding personalized medicine [8, 9], and scaling optimization problems in healthcare [10].

Quantum machine learning (QML) has more recently joined the AI/ML and quantum computing fields. The co-design of QML algorithms and hardware has led to early successes in the training and deployment of quantum Boltzmann machines [11], quantum boosting algorithms [12], and quantum classifiers [13] on quantum annealers. The availability of gate-based quantum platforms that support trainable unitary operations has led to the development of variational hybrid algorithms that harness quantum models with classical optimization and training workflows [14, 15]. It’s been shown that these parameterized quantum circuits can be trained as binary classifiers using the qubit readout [15, 16]. Quantum binary classifiers can even be constructed using a single qubit, though in practice the number of qubits used is related to the number of classical features that can be embedded into the quantum states [15, 17].

Currently scaling a variational hybrid workflow to train a QML model using hardware measurements faces certain bottlenecks due to the number of circuits, circuit depth, and measurements needed for implementation. Ensemble machine learning methods can potentially alleviate some of these bottlenecks by training a collection of weak learners: models that are under-parameterized and unable to capture highly complex relationships between features and outputs. Aggregation over multiple models and data is a well-established statistical method [18], and has led to machine learning ensembles built via bagging [19] and boosting [20]. This has carried forward to more modern ensemble techniques such as XGBoost [21], LightGBM [22], and CatBoost [23]. Such methods are appealing when mapping QML applications onto near-term quantum hardware as weak learners can be implemented with smaller, shallower unitary circuits.

More broadly, quantum machine learning and quantum ensemble techniques offer a complementary modeling paradigm that may be particularly well suited to regimes characterized by high dimensionality, limited labeled samples, and constrained model capacity [24, 25]. Ensemble constructions, either implemented classically over independently trained quantum models or natively within quantum representations, offer a principled mechanism for improving robustness, reducing variance, and mitigating trainability issues without resorting to deeper or more resource-intensive circuits [26, 27]. Importantly, these potential benefits do not rely on asymptotic quantum advantage, but rather on near-term characteristics such as circuit diversity, stochastic measurement noise, and alternative aggregation schemes.

There have been several demonstrations of ensembles of quantum learners [28–30]. Quantum boosting and bagging approaches have been developed for adiabatic [12] and gate-based quantum systems [31]. For quantum boosting there has been promising signs of trainability and generalization performance [32]. In this study, we chose to model immunotherapy response prediction in two renal cell carcinoma (RCC) cohorts using gene expression data [33, 34]. While gene expression data has been valuable in modeling response to immunotherapy in certain cancers, it has not translated well to RCC. Here we empirically compare the performance of different quantum ensembling approaches on this sample-limited data in simulation and on quantum devices. We evaluate the performance and overhead of classical aggregation (boosting, bagging, and soft-voting) and quantum aggregation (bagging via superposition and perturbation) methods in predicting immunotherapy response.

## Methods

In this work, we are focused on ensemble learning methods, a subset of supervised learning, where rather than train a single classifier, multiple classifiers, whose performance individually may be suboptimal, are aggregated through various methods to yield improved predictions. Here, we are training ensemble classifiers to predict binary labels. We leverage standard supervised learning, using datasets of labeled multi-dimensional features $\lbrace (X_{i}, y_{i}) \rbrace$. We evaluate several constructions of quantum ensembles: using classical aggregation via soft voting, bagging [19], or boosting [20]; and quantum bagging and perturbation [31]. These ensembles are distinguished by the methods used to aggregate predictions from each individual learner, and the feature partitions on which each learner trains.

### Quantum ensembles of quantum cosine classifiers

In this study we apply an implementation of a quantum cosine classifier (QCC) that serves as the base learner for a quantum ensemble cosine classifier (QEC) from [31]. In Macaluso *et al.*, they developed a bagging ensemble strategy where the quantum algorithm evolves the input state into a superposition of multiple quantum trajectories, effectively generating $2^{d}$ transformations after $d$ steps as captured by $d$ control registers. A quantum classifier, here a QCC, then makes classifications across these trajectories, and predictions are averaged together for a final ensemble prediction measured with a single qubit. This QEC approach offers important benefits as compared to other classical and quantum ensembling techniques, including ensemble sizes that scale exponentially with only linear scaling of depth and access to large ensembles from relatively few qubits owing to the final prediction being taken from the measurement of a single register. Further, the quantum computing properties of entanglement, superposition, and interference reduce the overhead of state preparation to be the equivalent of a single classifier and the actual classification performed with a single circuit execution as it propagates to all trajectories at once [31].

#### Quantum cosine classifier

The QCC, which serves as the weak learner for the QEC, uses a swap-test [35] to calculate the cosine distance of two sample vectors in a quantum state via interference. For a given test sample, it returns the probability of a sample belonging to a class from a single-qubit measurement based on its distance from a randomly selected training sample. The probability of a test sample $x_{test}$ belonging to the $x_{}$ sample class is given by


(1) \begin{eqnarray*}& Pr(y_{test} = y_{train}) = \frac{1}{2}+\frac{[D(x_{train},x_{test})]^{2}}{2}\end{eqnarray*}

where D(.,.) is the cosine distance between $x_{train}$ and $x_{test}$.

**Figure 1:**
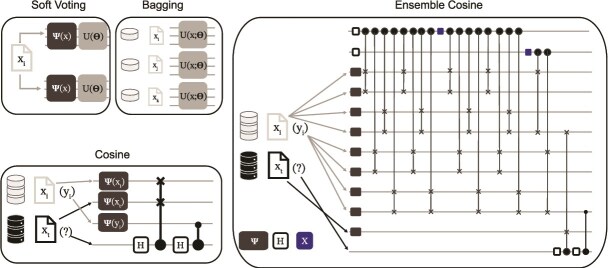
Comparison of quantum ensemble constructions. Two variational ensembles (Soft Voting and Bagging), the cosine classifier, and the quantum ensemble cosine classifier are shown. In the variational ensembles each learner predicts the label of one encoded feature vector $x_{i}$ at a time using a parameterized unitary. In the Soft Voting panel we show the unitary construction of the variational classifier—which we condense into $\mathcal{U}(x;\theta )$ in the remaining panels. Cosine classifiers predict an unknown label using multiple encoded feature vectors $x_{i}, x_{t}$ and also the known label information $y_{i}$. The quantum ensemble cosine classifier has ancilla qubits for the control register (top two qubits here). In this is example four unique training samples are used to predict a single test sample. The non-variational ensembles do not have trainable parameters. During variational training samples are drawn from either the entire training dataset and used to train all learners (Soft Voting), or the dataset is partitioned into non-overlapping subsets and each learner only has access to a specific subset (Bagging). Boosting (not shown) uses random weighted subsets.

The QCC circuit encodes the training data, training label, and test data into three qubits represented in amplitude-encoded states with an additional qubit to store the prediction (Fig. 1). The swap test transforms the amplitudes of the test qubits as a function of the squared cosine distance [31]. The learning via interference is achieved through a Pauli-$X$ rotation applied with the training label as the control qubit thereby leaving the test amplitude the same if of class 0 and inverting if of class 1, with the probability the test sample is class 1 increasing as the similarity between the train and test vectors grows. This classifier is well suited as a weak learner because of the high variance subject to the random selection of the training sample. The QCC uses one training sample and two features, yielding circuits requiring four qubits.

#### Quantum ensemble cosine classifier

The QEC uses a quantum circuit to capture independent quantum trajectories by sampling in superposition from $2^{d}$ transformations of the training set and then averaging across the predictions. After this sampling, learning via interference proceeds as defined in the QCC. While we refer the reader to [31] for an in-depth description of the method, we provide a brief summary below. The QEC utilizes three quantum registers: data register for training data; test register for test data; and control register for $d$ control qubits, which dictates the number of transformations of the training data that will exist in superposition.

The QEC circuit (Fig. 1) performs the four key components of the algorithm: state preparation, sampling in superposition, learning via interference, and measurement. State preparation is performed with a Walsh–Hadamard gate on the control register and amplitude encoding of the data and test registers. Sampling in superposition is achieved through entangling the *i*th control qubit with two transformations of $|x,y\rangle$ using two random unitaries, $\mathcal{U}_{(i,1)}$ and $\mathcal{U}_{(i,2)}$, and a Pauli-$X$ gate for $i=1,...,d$. This yields a fully entangled stated with $B=2^{d}$ quantum trajectories. Learning via interference is performed using a quantum classifier, here the QCC, which predicts the class of a test sample for a given quantum trajectory of the original training data. Finally, measurement is performed on the test register to capture the average prediction of the classifiers. We modified the original implementation to enable increased training sample sizes and number of features considered. We tested multiple configurations over a range of parameter values: $d=[1,2,3]$, $n{\_}train=[2,4,8]$, $n{\_}swap=[1,2,4]$, and $n{\_}feature=[2,4,8]$. These configurations yield circuits whose qubit requirements range from $7-56$ executed with 8192 shots.

#### Quantum ensemble cosine classifier with random unitaries

While the procedure above yields unitary transformations that are uncorrelated in general, a natural question to ask is what effect on performance is there if using other forms of random sampling on $\mathcal{U}(n)$. This opens up essentially all distributions on $\mathcal{U}(n)$ as possible choices for sampling. We introduce here a quantum ensemble cosine classifier with randomly sampled unitaries (QECRU), though given that an exhaustive analysis of all such possible choices is beyond the scope of this work, we focus on the choice with the fewest assumptions: the uniform distribution on $\mathcal{U}(n)$, meaning we are considering the unique measure invariant with respect to group multiplication. We use the function scipy.stats.unitary_group (*v1.6.1*) to generate as many different random unitary operators as required. The implementation follows [36], and samples a standard normal random complex matrix, decomposes it with QR decomposition, and scales the orthogonal factor by an entry-wise normalization of the diagonal of the upper triangular factor. Using the same circuit from the QEC and replacing $\mathcal{U}_{(i,1)}$ and $\mathcal{U}_{(i,2)}$ with a randomly sampled $\mathcal{U}(n)$, we tested multiple configurations over a range of parameter values: $d=[1,2,3]$, $n{\_}train=[2,4]$, $n{\_}swap=[1,2,4]$, and $n{\_}feature=[2,4,8]$. These configurations yield circuits whose qubit requirements range from $8-23$ and executed with 8192 shots.

### Variational quantum ensembles

Variational quantum classifiers [15, 16] translate classical supervised learning into hybrid workflows. Label predictions $\hat{y}_{i}$ are made using finite samples sampled from quantum states prepared using parameterized quantum circuits ($\mathcal{U}(x_{i},\theta )$). Many approaches found in the literature use the expectation of a fixed observable to predict class labels. Our approach uses the observed occurrence of the (0/1) bitstrings when qubit 0 is measured in the computational basis. With this approach it is straightforward to extract class probabilities $p(y=0), p(y=1)$ and to train using binary cross entropy loss. Details on the training workflows are provided in the Supplementary Material.

The “weak learners” of our variational ensemble are shallow-depth parameterized quantum circuits. From the large design space of parameterized quantum circuit ansatzes, we use specific design choices and constraints. We choose amplitude embedding to map a multi-dimensional feature $x_{i}$ into the $2^{n}$-dimensional Hilbert space of $n$-qubits. Second, a parameterized single qubit rotation, decomposed as a RZ-RY-RZ gate sequence, is applied to each qubit (3 independent trainable parameters per qubit). If the learner has more than two qubits, this is followed by a layer of CNOT gates applied between qubits $(i, i+1)$. Third, another parameterized rotation is applied to each qubit, followed by classical readout of qubit 0 that is post-processed to make a label prediction.

A variational learner on $n$ qubits will have $6n$ trainable parameters, and an ensemble of $k$ learners has a total $6nk$ parameters to train. The variational ensembles of classifiers are trained using mini-batch gradient descent with Adam [37] and, using parameter shift rules to evaluate analytic circuit gradients [38]. We optimize hyper-parameters using k-fold cross-validation (k=4) and a grid of 90 configurations: three Adam learning rates ($\alpha \in [1 \times 10^{-3}, 1 \times 10^{-2}, 1 \times 10^{-1}]$), five batch size ($b \in [1, 2, 4, 8, 16]$), and seven ensemble sizes ($n_{\ell } \in [1,2,3,4,5,6,7]$). We used the Gaussian blobs and use the validation set performance to down-select on optimal ensemble designs, which are re-fit on the full training dataset. In particular, the datasets with overlapping blob centers $p1=p2$ under amplitude encoding, will see all features mapped close to the equator of the Bloch sphere, where the output probabilities $p(y=0) \approx p(y=1) \approx \dfrac{1}{2}$, and with smaller $cluster{\_}std$ the encoded features will be located in a narrower band around the equator.

#### Soft voting variational classifier

Every learner generates a predicted class membership based on the probability of observing the single qubit bitstrings $0/1$ in the $|0\rangle$ or $|1\rangle$. The final state label is predicted by aggregating the probabilistic output of sampled outputs using the average of all predictions $\dfrac{1}{K}\sum _{k} p^{k}(y_{i}=1)$. This workflow relies on serial processing of all samples. For soft-voting with blob datasets with well-separated cluster centers (e.g. $p1= 0.3, p2=1.$ and $p1=0.3,p2=0.5$ with either $cluster{\_}std=0.3, 0.5$), we observe that all ensemble sizes, all batch sizes trained with learning rates $\alpha =1 \times 10^{-2}, 1 \times 10^{-1}$ could achieve median validation set accuracy of $70\%$ or higher, using learning rate $\alpha =1 \times 10^{-3}$. For blob datasets with overlapping centers ($p1 = p2 = 1, cluster{\_}std = 0.5$), or ($p1 = p2 = 0.5, cluster{\_}std = 0.3$), only a few ensemble configurations could achieve median validation set accuracy above $50\%$. We choose to retrain ensembles containing up to 4 learners, including a single classifier as a control. With two-dimensional features (1 qubit per learner) these ensembles require a maximum of 8 qubits to instantiate the ensemble. However for the RCC data, with 3 qubits required per learner, the circuit size grows to 12 qubits. The re-training used learning rates $\alpha = [1 \times 10^{-3},1 \times 10^{-1}]$, and the same configurations (and learning rates) are used to train on the RCC data.

#### Bagged variational classifier

A bagged ensemble of [k] classifiers is trained by first partitioning the training data into [k] distinct subsets. The k-th learner is only trained on the k-th data subset. During the inference stage all learners make a prediction, which is aggregated using a weighted mean over each learners’ prediction $p(y=1)$. For bagged ensembles, the blob datasets with well-separated cluster centers were easiest to learn—with learning rates $\alpha =1 \times 10^{-2},1 \times 10^{-1}$ all ensemble configurations could achieve median validation sets accuracy near $100\%$ while for $\alpha =1 \times 10^{-3}$ smaller ensembles and smaller batch sizes performed better. For blob datasets with overlapping cluster centers and smallest $cluster{\_}std$, three configurations were able to achieve median validation accuracies above $62\%$: $(b, n_{\ell }) = [(2,3),(4,6),(8,3)]$. We take these three configurations and re-train them on the Gaussian blob datasets using $\alpha = [1 \times 10^{-3},1 \times 10^{-1}]$. The same configurations (and learning rates) are used to train on the RCC data.

#### Boosted variational classifier

A boosted ensembles of variational classifiers can use AdaBoost [39, 40] or general gradient boosting [41]. Our boosting implementation iteratively updates the weight (importance) of individual samples in the training set. Training samples are initially equal weighted and a weak classifier is trained on a random subset (drawn without replacement). The weak learner’s error on the entire training set is used to update the sample weights, then a new learner is trained on a different training subset using the updated weights. For blob datasets with overlapping cluster centers, boosting was not able to train any ensemble that had a median validation accuracy above $50\%$ (random guessing). Instead we retain the top three individual configurations: $(b, n_{\ell }) = [(2,3),(4,6),(8,3)]$ and re-train this on the Gaussian blob datasets using $\alpha = [1 \times 10^{-3},1 \times 10^{-1}]$, and use the same configurations and learning rates to train on RCC data features.

### Classical ensembles

Classical ensembles are represented by random forests (RF) [42] and extreme gradient boosting (XGBoost) [21]. RF is a popular ensemble method for both classification and regression problems that extends the bagging technique with added randomness to enhance diversity among decision trees [42]. In brief, RFs utilize decision trees from random subsets of samples and features to induce independence between trees. Voting is traditionally a “majority rule”. RF is robust and adept at handling high-dimensional datasets with complex interactions with demonstrated advantages on tabular data compared to newer deep learning methods [43]. Yet, RF can be challenged by small datasets and highly correlated features. We applied RF classifiers using *scikit-learn* (*v1.6.1*) with parameter optimization using the *RandomizedSearchCV* function from *scikit-learn* testing parameter ranges found in Table 1.

**Table 1:** Hyperparameter values for Random Forest

Parameter	Values
$n{\_}estimators$	$[100,\ldots ,1000]$ by step 100
$max{\_}depth$	$[5,\ldots ,20]$
$min{\_}samples{\_}split$	$[2,\ldots ,10]$
$min{\_}samples{\_}leaf$	$[1,\ldots ,5]$
$max{\_}features$	{sqrt, log2}

XGBoost is a widely used supervised learning method performing gradient boosting. It builds decisions trees sequentially, where each tree corrects the errors of the ensemble of preceding trees. It incorporates L1 and L2 regularization to avoid overfitting together with a differentiable loss function. XGBoost is a scalable architecture that includes tree pruning, sparsity aware learning, and cache-aware access. XGBoost often generalizes better than RF though is more sensitive to hyperparameters. We applied XGBoost using the *XGBClassifier* function from *xgboost (v3.0.0)* with parameter optimization using the *GridSearchCV* function from *scikit-learn* (*v1.6.1*) parameter ranges found in Table 2.

**Table 2:** Hyperparameter values for XGBoost

Parameter	Values
$n{\_}estimators$	$\{100,200\}$
$max{\_}depth$	$\{3,5,7\}$
$learning{\_}rate$	$\{0.01,0.1,0.2,\}$
$subsample$	$\{[0.7,0.8,1.0\}$
$colsample{\_}bytree$	$\{[0.7,0.8,1.0\}$
$min{\_}child{\_}weight$	$\{1,3,5\}$

### Datasets and feature selection

#### Gaussian blobs

**Figure 2:**
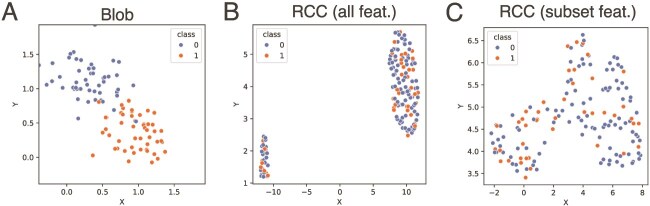
UMAP projections of study datasets. (A) Example of one tested configuration of Gaussian blobs. In this configuration, 100 samples are evenly drawn from two classes, class 0 and 1. Class 0 center coordinates are $(0.3,1.0)$ with $\sigma =0.3$, class 1 coordinates are $(1.0,0.3)$ with $\sigma =0.3$. (B–C) McDermott RCC datasets are projected using all gene features (B) and 8 gene features selected from literature (C).

The Gaussian blob benchmark is a synthetic dataset that was generated in *scikit-learn* (*v1.6.1*). Eighteen different configurations are used to create sets of two-dimensional features sorted into two classes over all combinations of the following parameters: $cluster{\_}std=[0.3,0.5]$, $p1=[0.3, 0.5, 1.0]$, and $p2=[0.3, 0.5, 1.0]$. $p1$ and $p2$ represent the x and y coordinates of the centers of class 1 ($p1$, $p2$) and class 2 ($p2$, $p1$) blobs. The features are generated in the domain $[-0.85, 2.55] \times [-0.85, 2.55]$ and are rescaled to [0, 1] using min-max scaling without standardization. The blob datasets contain 100 labeled samples and 10 unique 80/20 train/test splits are generated (Fig. 2). With only two-dimensions, no feature selection was required to enable quantum execution on simulation or hardware.

#### Renal Cell Cancer (RCC)

Using data from IMmotion150, a randomized phase 2 study that includes patients with treatment-naive metastatic renal cell carcinoma from McDermott *et al.* [33], we normalized RNA-seq mRNA counts for 150 patient samples generated from whole-transcriptome sequencing analysis using DESeq2 [44]. We also applied variance stabilizing transformation from DESeq2 to ensure constant variance across the range of mean values seen per sample. For analyses, we considered two feature sets, one with the entire gene feature space with 57,696 genes, and a curated subset of 8 genes (*CD8A, CXCL9, CXCL13, IFNG, CD274, PDCD1, VHL, GZMK*), which are known to associate with immunotherapy response [45, 46]. These genes were selected based on their established role as biomarkers for immunotherapy response and renal cell carcinoma prognosis. CD8A, CXCL9, CXCL13, IFNG, GZMK, PDCD1, and CD274 are related to lymphocyte activity within the tumor and immune activation. VHL loss is a hallmark driver of RCC, promoting tumor progression. Together, these genes represent a well informed set with likelihood of contributing to prognosis and response to immunotherapy in RCC.

All features are rescaled to $[0, 1]$ using a min-max scaling without standardization. Given the current constraints on circuit size for execution on quantum simulation and hardware, dimensionality reduction was necessary when analyzing the entire gene feature space. We performed principal component analysis (PCA) using *scikit-learn (*v1.6.1*), and the top $f$ principal* components by variance were used as features where $f$ was an experimental parameter. For the classical ensembles, this dimensionality reduction was not performed as they are not subject to the same current computational constraints. The patients in the dataset are categorized as Responders and Non-responders based on Response Evaluation Criteria in Solid Tumors v1.1 (RECIST v1.1). Individuals exhibiting either a Complete Response or Partial Response were classified as Responders, while those with Stable Disease or Progressive Disease were categorized as Non-Responders. The RCC dataset’s 150 samples were divided into 10 train/test splits (80/20). We visualize the datasets using two-dimensional features extracted using the uniform manifold approximation and projection (UMAP) algorithm [47].

#### RCC validation cohort

Transcriptomic data for validation were obtained from a previously published cohort, Miao *et al.* [34] (PMCID: PMC6035749), of 33 patients with metastatic renal cell carcinoma (mRCC) treated with immune checkpoint inhibitors. mRNA count data were normalized and variance stabilized using DESeq2, consistent with the approach applied to the IMmotion150 dataset. Associated clinical annotations, including treatment response, were incorporated for downstream analyses similar to the McDermott *et al.* cohort.

## Results

### Classifier performance

For each model described in the Methods Section, we compared the trained ensemble performance on Gaussian blob data and RCC data. We assess the performance of our models using accuracy, weighted F$_{1}$ score from *scikit-learn*, and the Brier score $S = \langle (p_{i} - y_{i})^{2}\rangle$. The probability of outcome $y_{i}$ used in the Brier score is defined by the number of shots observed in the 0 or 1 bitstring. For accuracy and weighted F$_{1}$ metrics, higher scores indicate better predictions. For the Brier score, lower scores indicate better predictions. For each dataset configuration, we report the maximal mean performance over all data splits.

**Figure 3:**
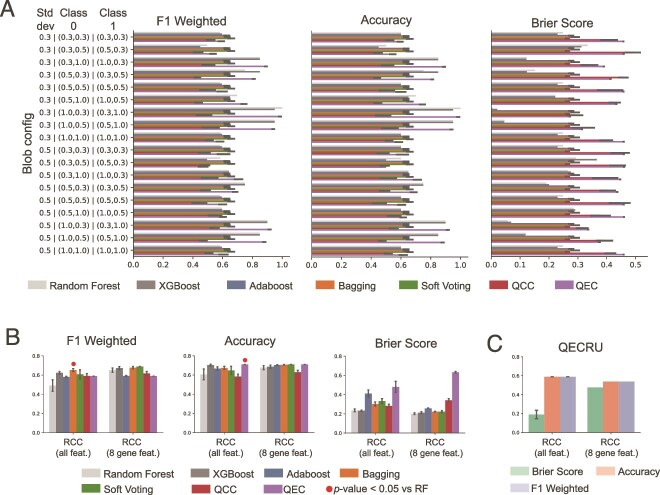
Comparison of ensemble classifier performance. Mean and standard error of each metric is calculated over splits for blob (A) and RCC (B) datasets for the RF, XGBoost, boosted variational ensemble, bagged variational ensemble, soft vote variational ensemble, quantum cosine classifier (QCC), quantum ensemble of quantum cosine classifiers (QEC), and QEC with random unitaries (QECRU). A) Performance per blob configuration for a given classifier configuration over 10 splits. Blob configurations are indicated along the y-axis with specified standard deviation and *x,y* coordinates for centers of class 0 and 1. For a given blob configuration and classifier type, we plot the classifier configuration that achieves the maximum mean performance. For ease of visualization, indicators of significantly improved performance by quantum ensemble methods are omitted. All statistical comparisons for blob datasets can be found in Supplementary Table 1A. B) Performance of classifiers on RCC datasets over 10 splits, applied to all available features and a curated 8 gene feature subset. For quantum ensembles applied to all available features, PCA was employed for dimensionality reduction. Red circle indicates significant improvement over RF calculated by $t$-test. There were no instances of XGBoost being significantly outperformed. C) Performance of QECRU over five splits. All statistical comparisons for McDermott datasets can be found in Supplementary Table 1B.

Across the 18 blob configurations (Fig. 3A, Supplementary Table 1A), RF, XGBoost, and QEC perform similarly when considering the accuracy and F$_{1}$, and both outperform the QCC. QCC reached an F$_{1}$ or accuracy $\approx 0.5$, despite blob configurations that were well separated, as may be expected from being a single weak learner. The variational bagging, boosting, and soft voting classifiers were comparable in many instances, though there were numerous examples where the RF, XGBoost, and QEC significantly outperform the variational methods and the majority of quantum ensembles with significant improvement over RF or XGBoost were QEC (Supplementary Table 1A).

On the complete McDermott RCC dataset (Fig. 3B,C, Table 4 and Supplementary Table 1B), we found most quantum classifiers showed comparable to slightly improved F$_{1}$ and accuracy scores as the RF, with the exception of the bagging variational classifier with significantly improved F$_{1}$ score ($P$-value = .009 by one-sided $t$-test) and QEC with significantly improved accuracy ($P$-value = .041). While compared to XGBoost the quantum ensemble models did not achieve significantly better performance, however, QEC and the bagging variational methods were comparable. Among the quantum methods, when we identified the maximal performance for any data split, we found the bagging classifier reached the highest F$_{1}$ of 0.81 (Table 4). When testing on the RCC dataset with 8 selected gene features, the quantum and classical models generally had comparable performance.

**Table 3:** Parameter values and quantum resources for quantum hardware experiments

Parameter	HW1	HW2	HW3	HW4
Samples	2	8	8	8
Features	2	32	32	16
Control Registers	1	2	2	2
Random Swaps	1	1	1	1
Qubits	7	56	56	47
Total Depth	100	853	853	541
2-qubit Depth	20	201	201	163
Error mitigation	PT	PT	PT & DD	PT & DD

**Table 4:** Comparing best performing ensembles on an individual RCC test dataset split using a PCA feature embedding from the full feature space, and reporting on circuit width, circuit depth including measurement, feature size, and weighted F$_{1}$ score

Model	Features	Qubits	Depth	2Q depth	F$_{1}$
Cosine	2	4	64	11	0.77
Variational	8	3	59	14	0.78
Ensemble Models					
QEC	4	16	132	27	0.72
QECRU	8	8	143	30	0.66
Soft Vote	4	10	22	12	0.78
Bagging	8	9	57	14	0.81
Boosting	2	6	6	0	0.68

Given the protracted execution times to simulate the QECRU, we report its performance over 5 splits of the data (Fig. 3C), and find it to perform at par with the QEC, albeit with an improved Brier score. We also observe that on the complete RCC dataset, RF predicted only a single class for 9 of the 10 splits. This undesired behavior was absent when using the QCC, and appeared to lesser degrees for XGBoost, QEC, and QECRU with only 3/10, 4/10, and 2/5 splits predicted as a single class, respectively. For those splits where the classifiers were not pathologic, we observe similar mean performance values for the RF ($F_{1}=0.52$), XGBoost ($F_{1}=0.67$), QCC ($F_{1}=0.61$), QEC ($F_{1}=0.58$), and QECRU($F_{1}=0.60$).

**Figure 4:**
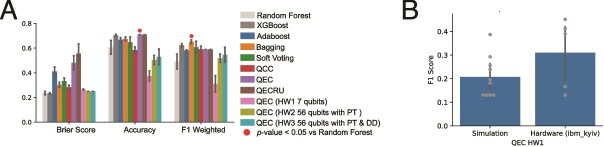
Comparing simulation and quantum hardware QEC performance. A) Performance of the QEC executed on quantum hardware, $ibm{\_}kyiv$ with 7 and 56 qubits are shown with settings in Table 3. These classifiers were run over five splits of the RCC data with PCA embeddings with 2 and 32 principle components as features for the 7 and 56 qubit experiments, respectively, using either Pauli twirling (PT) or PT and dynamical decoupling (DD) error mitigation. Classifiers shown in Figure 3B,C are included for ease of comparison. Mean and standard error of each metric is calculated over splits for RCC datasets. Red circle indicates significant improvement over RF calculated by $t$-test. There were no instances of XGBoost being significantly outperformed. B) Comparison of QEC executed with configuration HW1 (Table 3) on simulator and hardware (*ibm_kyiv*). There is not a significant difference in performance between simulation and hardware as calculated by a $t$-test ($P$-value = .111).

Further, we performed experiments on a 127 qubit IBM Eagle R3 quantum processing unit (QPU) $ibm{\_}kyiv$, using QEC on the McDermott RCC dataset over five splits using configurations based on well performing models from simulation (Fig. 4A and Table 3). These experiments were performed using Qiskit SamplerV2 primitive with 8192 shots and error mitigation including Pauli twirling (PT) and dynamical decoupling (DD) with *XY4* gate sequence. Circuits were transpiled using Qiskit’s in-built transpiler with optimization level 3 (See Section *Overhead*) The first experiment (HW1) using 7 qubits significantly underperformed simulator experiments. We then ran a larger 56 qubit circuit (HW2 and HW3) with different levels of error mitigation. With only PT, the configuration underperformed QEC in simulation. With PT and DD error mitigation, performance improved and reach a similar weighted F$_{1}$ to QCC, QEC, and QECRU, which is slightly improved to the RF though underperformed XGBoost.

We examined the amount of performance degradation with moving from simulation to hardware for an individual QEC configuration (HW1) that could be simulated and found there to be no significant decrease in performance, in fact there was a modest increase in the median F1 score (Fig. 4B). Though considering experiments with larger circuits on hardware (HW2 and HW3) where the impact of hardware noise can be more strongly felt, the accuracy significantly decreases as compared to the best performing QEC simulation, though the weighted F1 is more comparable (Fig. 4A). Some measure of this performance degradation may be mitigated by more advanced error mitigation techniques or execution of more advanced QPUs, such as the latest generation IBM Heron R3 or Nighthawk chips.

**Figure 5:**
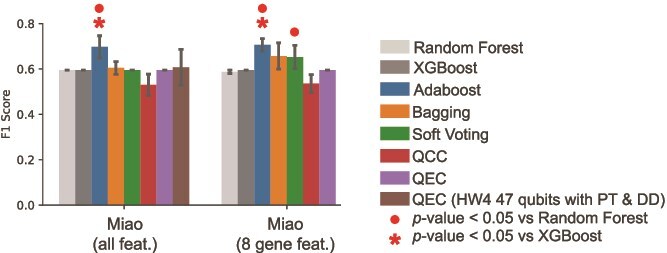
Quantum ensemble performance on Miao *et al.* RCC data. Mean and standard error of each metric is calculated over 10 data splits for the RF, XGBoost, boosted variational ensemble, bagged variational ensemble, soft vote variational ensemble, quantum cosine classifier (QCC), and quantum ensemble of quantum cosine classifiers (QEC). An additional QEC (HW4) was executed on an IBM Heron R2 QPU (*ibm_cleveland*) at 47 qubits with PT and DD error mitigation. Red circles and asterisks indicate significant improvement over RF and XGBoost, respectively, calculated by a $t$-test.

We performed validation experiments using a separate RCC dataset from Miao *et al.* [34]. This data of 33 patients was analyzed in the same fashion as the McDermott *et al.* data, and we observed similar performance characteristics for both the variational and cosine quantum ensembles (Fig. 5). Soft voting quantum ensembles outperformed RF, and boosted ensembles surpassed both RF and XGBoost. We again tested QEC on quantum hardware using a 47 qubit circuit (HW4) similar to HW3 (Table 3) though using only 16 PCA components rather than 32, a result of the reduced training cohort size in this smaller dataset. The QPU for these experiments, *ibm_cleveland* was a newer 156-qubit IBM Heron R2 with lower error rates, faster gate speeds, and near elimination of cross-talk as compared to the Eagle R3 QPU. QEC experiments on Heron R2 QPU were comparable to noise free simulation of quantum ensembles, as well as the classical baselines (Fig. 5).

## Discussion

### Comparison to classical baseline

To better understand the effect of qubit overhead, embedding structure, and feature structure, we focus on performance from simulation where more extensive experimentation was performed. With the synthetic dataset, variational approaches slightly outperformed the RF and cosine classifiers for many of the blob configurations. However for blob configurations whose centers were well-separated ($(1.0,0.3)$ and $(1.0,0.5)$), the RF, XGBoost, and QEC were notably out-performing all others. It would be expected that configurations with well separated classes would see higher predictive performance from all methods, yet the performance of the variational ensembles are relatively consistent across all configurations. While it has been shown that universal classifiers can be constructed with a single qubit [48], the variational ensembles are composed with parameterized circuits of limited depth. This attempts to balance trainability (ability to learn nonlinear decision boundaries) while working to avoid over-parameterization and over-fitting.

In contrast for the significantly more complex RCC datasets, we find the variational methods performing well with the bagging classifier significantly improving on the RF in both RCC datasets and outperforms XGBoost in the Miao dataset. The other quantum classifiers were doing well compared to RF with a modest, though insignificant, increase in F$_{1}$ or accuracy by $\approx 10\%$. While the performance of QEC and QECRU was not generally significantly improved over the variational and classical ensemble classifiers, they were able to achieve this performance using only 2-4 training samples in simulation and 8 samples on a quantum device. This suggests the great potential for applications where the amount of data is highly constrained and methods still need to effectively learn.

Though the performance of the variational ensembles were comparable to the QEC, the variational ensemble sizes were limited to fewer than 10 learners owing to the expensive hyperparameter grid search. Therefore variational ensembles fully trained on the RCC data used more samples to train each learner: soft voting used all training samples per learner, bagging used $|X|/\ell$ samples per learner, and boosting also used $|X|/\ell$ samples per learner. Variational ensembles could be trained using fewer samples per learner if the number of learners in the ensemble was increased to $\ell> 25$. Therefore both variational and cosine quantum ensembles share this value of being able to learn using fewer samples with broad applications to data-constrained problems.

### Overhead

To assess the utility of our approaches, we consider their overhead and scaling (qubit overhead, gate depth). The QEC and QECRU utilizes a parallelization in feature processing enabled by its $2^{d}$ transformations of the input state with linear cost in circuit. These classifiers’ qubit overhead scales logarithmically with the feature size and linearly with the number of training sample size and number of control registers. Transpiled circuit depth though grows rapidly with register size, number of swaps, and training size, particularly with the prevalence of long distance CNOT gates. Using the maximum available transpilation optimization in Qiskit (level 3) on a quantum circuit with 56 qubits acting on 32 features, 2 control registers, 8 training samples, and 1 random swap, the total and 2-qubit transpiled depths were 853 and 201, respectively (Table 4). While this optimization level includes layout aware optimization, optimal qubit selection, SWAP gate minimization, Cartan KAK Decomposition [49] to resynthesize 2-qubit blocks, and unitary breaking passes, further reduction of circuit depth may require more careful construction of the ansatz or utilization of advanced circuit knitting techniques [50]. For similar sized circuits from HW4 performed on the validation dataset, QPU execution time was $\approx{4}s$ on *ibm_cleveland* with 8192 shots, but could not be exactly simulated in statevector simulation owing to the exponential growth in memory requirements.

The feature scaling of the base variational classifier is the same in qubit overhead, using $\log ({f})$ qubits to encode $f$ classical features, this is due to our choice of amplitude encoding. While this makes comparisons between the different quantum ensembles tractable, this approach produces deep circuits that are not robust to hardware noise. There are several aspects of the variational training that have poor scaling. First, the dependence on supervised training makes them less efficient to scale up as our models process one sample at a time. Second, the use on gradient-based optimization incurs high circuit overhead for each gradient estimation [51].

For the bagged and boosted ensembles, the learners are trained one at a time on disjoint subsets and ensemble predictions are aggregated only during inference. This made the numerical simulation of bagged and boosted ensembles quite fast as each learner was constructed and trained one at a time—for the largest feature size of $8$ this required at most $3$ qubits and $18$ trainable parameters. The bagged ensemble training could be easily distributed, but not the boosted ensemble training due the adaptive weighting of the training samples. On the other hand, the soft-voting ensemble was the slowest to train and incurred the highest simulation overhead. These ensembles used the highest amount of memory during training and the gradient update step was a major bottleneck and this is wholly due to how the soft-voting learners were updated during training.

Implementing the gradient update for each learner and ensemble could be done in multiple ways. For the soft-voting ensemble, the ensemble predictions are needed to evaluate the loss at each step of gradient descent. We implemented the soft-voting ensemble training without distributing the execution of each individual learner and generated the ensemble predictions by sampling from the state prepared in a single circuit of $n \times \ell$ qubits: $\Psi = \mathcal{U}_{0} \otimes \mathcal{U}_{1} \otimes \dots \mathcal{U}_{\ell }$.

The trained variational ensembles were not deployed on hardware, and this poses an open question about the number of quantum resources each ensemble needs. The aggregation of the learner predictions is implemented classically as a post-processing step. Thus to generate predictions from an ensemble of $\ell$ learners we can execute each learner serially using a dedicated $n$ qubit state preparation and measurement circuit for each. However this does not seem like an efficient use of near-term quantum processors, which offer far more qubits than the number used per learner in this study. The second approach would be to simultaneously prepare all $\ell$ learner states using disjoint hardware qubit subsets. However, as the register size increases the sampling overhead will also increase. Additionally, when executed on hardware the assumption that each learner will remain independent and unperturbed by the gates applied to other learners is dependent on the presence of correlated noise.

## Conclusions

Quantum ensemble models are a potential path to utility scale QML applications: replacing quantum deep learning approaches with quantum methods closer to RFs or using random subspace sampling to extract data to train weak learners. The potential to harness quantum superposition as an inherent parallelization is a particularly attractive feature of some of these methods. The QEC is able to move through $2^{d}$ quantum trajectories and take a single measurement to obtain a prediction. This enables exploring a vast landscape with relatively few qubits, though with the need to balance circuit depth. While we demonstrate that both classical and quantum ensembles are able to learn from relatively few features as evidenced by the comparable performance when using all available features or the curated 8 cancer gene subset, we further show quantum ensembles can learn from fewer training examples, even in this reduced feature space.

The ability to learn from fewer training examples is highly sought after in HCLS applications where patient samples are typically difficult to acquire and problems are often under-determined. This sort of challenge is exemplified when new phenotypes of interest emerge. For example, a new viral strain is discovered and an ML model to predict susceptibility would rely on either transfer learning or be required to learn from the limited samples in this new exposure. Both scenarios are a challenge for classical ML. Therefore it is noteworthy that QEC and QECRU were often able to reach comparable performance to classical ensembles, which were trained on the entire training set, while using only 2-8 training samples, even in the case of a reduced 8 gene feature space. While the feature space reduction was performed using biological knowledge defined *a priori* to identify genes likely to have large effects in RCC, it could also have been performed using a more data driven strategy, such as selecting the top genes by variance.

Overall, this result on an actual HCLS problem, immunotherapy response prediction in RCC, highlights the importance of this approach to data-constrained problems. While we demonstrate this performance on two RCC datasets, the application of these quantum ensemble methods is not specific to this cancer or any specific type of data, and may be considered for any small-data biological data problems. This is in keeping with previously identified beneficial behavior of quantum machine learning methods over classical methods for problems with fewer training data [5]. This behavior combined with the ability to learn with few features, which here was shown as a characteristic of both classical and quantum ensembles, may uniquely position certain quantum ensemble methods for particular low data regimes, both low sample and dimension, meriting future exploration.

Utility-scale quantum computing typically requires large qubit registers (50+) to reach complexity unable to be simulated classically while still identifying signals in the presence of hardware noise and noisy unitary operations. Here we see a tradeoff between models that leverage superposition but requires ancilla qubits and long range connections, or variational models that require serial processing and parameterized gates. Our initial experiments deploying a 56 qubit QEC on a QPU (Fig. 4) demonstrates that with minimal error mitigation and only 8 training samples, the QEC was comparable to RFs and improved upon the performance of smaller QECs in noise-free simulation. This performance increase at 56 qubits from the earlier 7 qubit hardware experiment is significant and suggests that greater performance may be achieved with more expressive circuits. The QEC model can scale up to larger and more complex circuits by increasing the number of control registers, training samples, and features. To build towards utility-scale demonstrations, more complex circuits, additional error mitigation strategies, and execution on more advanced QPUs with greatly reduced noise or on eventual fault-tolerant quantum devices expected within the next 3–4 years are needed to move to problem scales beyond the reach of classical ensembles.

With the promising results of these initial experiments, we believe that this opens the way for the use of quantum ensemble approaches to model biomarkers of the immunotherapy response. These ensembles represent a new way forward in bioinformatics, integrating the strengths of classical ensemble learning with the unique advantages of quantum computing. Their ability to learn efficiently and generalize from limited data positions them as particularly valuable for under-determined or data-constrained problems. Furthermore, these methods lay a framework for combining different data modalities such as whole exome sequencing-based measurements of copy number changes and tumor mutational burden alongside gene expression. Capturing the multi-modal interaction of complex biological features is of great interest for clinical trial design and the identification of personalized medicine approaches. Given the prevalence of such problems in HCLS, quantum ensembles stand out as important tool worthy of deeper study to advance predictive modeling and accelerate discovery.

**Key Points**

- We provided a survey of variational and non-variational quantum ensembles for learning in healthcare and life sciences, highlighting advantages and challenges.
- We introduced a new extension of the quantum ensemble cosine classifier to incorporate randomly sampled unitaries.
- We demonstrated in both quantum simulation and experiments on quantum devices using up to 56 qubits that these quantum ensembles are able to achieve comparable to improved performance with respect to classical ensemble methods while using an order of magnitude fewer samples or weak learners in two separate RCC datasets.
- We show that quantum computing holds promise for data-constrained problems in healthcare and life sciences with are our findings and insights aimed at guiding future research using quantum ensembles, particularly for small data learning.

## Supplementary Material

Supplementary_Material_Bbag280

## Data Availability

The biological data underlying this article are available at the European Genome-Phenome Archive (EGA) under accession number EGAS00001002928. Code for the quantum cosine and quantum ensemble cosine classifiers can be found at github.com/IBM/QBioCode/tree/main/tutorial/QEnsemble. Pseudo-code for variational quantum ensembles has been provided in Supplementary materials.

## References

[1] Tarca AL, Carey VJ, Chen X-w et al. Machine learning and its applications to biology. *PLoS Comput Biol* 2007;3:e116. 10.1371/journal.pcbi.0030116 17604446 PMC1904382

[2] Cordier BA, Sawaya NPD, Guerreschi GG et al. Biology and medicine in the landscape of quantum advantages. *J R Soc Interface* 2022;19:20220541. 10.1098/rsif.2022.0541 PMC9709576 36448288

[3] Greener JG, Kandathil SM, Moffat L et al. A guide to machine learning for biologists. *Nat Rev Mol Cell Biol* 2022;23:40–55. 10.1038/s41580-021-00407-0 34518686

[4] Ott S, Barbosa-Silva A, Blagec K et al. Mapping global dynamics of benchmark creation and saturation in artificial intelligence. *Nat Commun* 2022;13:6793. 10.1038/s41467-022-34591-0 36357391 PMC9649641

[5] Caro MC, Hsin-Yuan Huang M, Cerezo KS et al. Generalization in quantum machine learning from few training data. *Nat Commun* 2022;13:4919. 10.1038/s41467-022-32550-3 35995777 PMC9395350

[6] Bose A, Rhrissorrakrai K, Utro F et al. Advancing single-cell omics and cell-based therapeutics with quantum computing. *Nat Rev Mol Cell Biol* 2026;27. 10.1038/s41580-025-00918-0 41478876

[7] Basu S, Born J, Bose A et al. Towards quantum-enabled cell-centric therapeutics. arXiv preprint, arXiv:2307.05734, 2023.

[8] Flöther FF, Blankenberg D, Demidik M et al. How quantum computing can enhance biomarker discovery. *Patterns* 2025;6:101236. 10.1016/j.patter.2025.101236 PMC12191739 40575130

[9] Emani P, Warrell J, Anticevic A et al. Quantum computing at the frontiers of biological sciences. *Nat Methods* 2021;18:701–9. 10.1038/s41592-020-01004-3 33398186 PMC8254820

[10] Doga H, Aritra Bose M, Sahin E et al. How can quantum computing be applied in clinical trial design and optimization? Trends Pharmacol Sci 2024;45:880–91. 10.1016/j.tips.2024.08.005 39317621

[11] Amin MH, Andriyash E, Rolfe J et al. Quantum Boltzmann machine. *Phys Rev X* 2018;8:021050. 10.1103/PhysRevX.8.021050

[12] Neven H, Denchev VS, Rose G et al. Qboost: large scale classifier training with adiabatic quantum optimization. In: Steven C. H. Hoi, Wray Buntine. eds. *Asian Conference on Machine Learning*, (Singapore Management University, Singapore), pp. 333–48. PMLR, 2012.

[13] Farhi E, Neven H. Classification with quantum neural networks on near term processors. arXiv preprint arXiv:1802.06002, 2018.

[14] Peters E, Caldeira J, Ho A et al. Machine learning of high dimensional data on a noisy quantum processor. *npj Quantum Inf* 2021;7. 10.1038/s41534-021-00498-9

[15] Havlíček V, Córcoles AD, Temme K et al. Supervised learning with quantum-enhanced feature spaces. *Nature* 2019;567:209–12. 10.1038/s41586-019-0980-2 30867609

[16] Schuld M, Bocharov A, Svore KM et al. Circuit-centric quantum classifiers. Phys Rev A. 2020;101:032308. 10.1103/PhysRevA.101.032308

[17] Schuld M, Sweke R, Meyer JJ. Effect of data encoding on the expressive power of variational quantum-machine-learning models. *Phys Rev A* 2021;103:032430. 10.1103/PhysRevA.103.032430

[18] Clark WAV, Avery KL. The effects of data aggregation in statistical analysis. *Geogr Anal* 1976;8:428–38. 10.1111/j.1538-4632.1976.tb00549.x

[19] Breiman L . Bagging predictors. *Mach Learn* 1996;24:123–40. 10.1023/A:1018054314350

[20] Freund Y, Schapire RE et al. Experiments with a new boosting algorithm. In: Lorenza Saitta, ed. ISML'96: Proceedings of the Thirteenth International Conference on International Conference on Machine Learning, Bari, Italy: Morgan Kaufmann Publishers, Vol. 96, pp. 148–56, 1996.

[21] Chen T, Guestrin C. Xgboost: a scalable tree boosting system. In: Balaji Krishnapuram, Mohak Shah, eds. Proceedings of the 22nd ACM SIGKDD International Conference on Knowledge Discovery and Data Mining, pp. 785–94, New York, NY: Association for Computing Machinery, 2016.

[22] Ke G, Meng Q, Finley T et al. Lightgbm: a highly efficient gradient boosting decision tree. *Adv Neural Inf Proces Syst* 2017;30:3149–57.

[23] Prokhorenkova L, Gusev G, Vorobev A et al. Catboost: unbiased boosting with categorical features. *Adv Neural Inf Proces Syst* 2018;31:6639–49.

[24] Schuld M, Killoran N. Quantum machine learning in feature Hilbert spaces. *Phys Rev Lett* 2019;122:040504. 10.1103/PhysRevLett.122.040504 30768345

[25] Huang H-Y, Broughton M, Mohseni M et al. Power of data in quantum machine learning. *Nat Commun* 2021;12. 10.1038/s41467-021-22539-9 PMC8113501 33976136

[26] McClean JR, Boixo S, Smelyanskiy VN et al. Barren plateaus in quantum neural network training landscapes. *Nat Commun* 2018;9:4812. 10.1038/s41467-018-07090-4 30446662 PMC6240101

[27] Cerezo M, Sone A, Volkoff T et al. Cost function dependent barren plateaus in shallow parametrized quantum circuits. *Nat Commun* 2021;12. 10.1038/s41467-021-21728-w PMC7979934 33741913

[28] Abbas A, Schuld M, Petruccione F. On quantum ensembles of quantum classifiers. *Quantum Mach Intell* 2020;2:1–8. 10.1007/s42484-020-00018-632879908

[29] Schuld M, Petruccione F. Quantum ensembles of quantum classifiers. *Sci Rep* 2018;8:2772. 10.1038/s41598-018-20403-3 29426855 PMC5807322

[30] Silver D, Patel T, Tiwari D. QUILT: effective multi-class classification on quantum computers using an ensemble of diverse quantum classifiers. *Proc AAAI Conf Artif Intell* 2022;36:8324–32. 10.1609/aaai.v36i8.20807

[31] Macaluso A, Clissa L, Lodi S et al. Quantum ensemble for classification. arXiv preprint arXiv:2007.01028, 2020.

[32] Wang Y, Wang X, Qi B et al. Supervised-learning guarantee for quantum adaboost. *Phys Rev Appl* 2024;22:054001. 10.1103/PhysRevApplied.22.054001

[33] McDermott DF, Huseni MA, Atkins MB et al. Clinical activity and molecular correlates of response to atezolizumab alone or in combination with bevacizumab versus sunitinib in renal cell carcinoma. *Nat Med* 2018;24:749–57. 10.1038/s41591-018-0053-3 29867230 PMC6721896

[34] Miao D, Margolis CA, Gao W et al. Genomic correlates of response to immune checkpoint therapies in clear cell renal cell carcinoma. *Science* 2018;359:801–6. 10.1126/science.aan5951 29301960 PMC6035749

[35] Buhrman H, Cleve R, Watrous J et al. Quantum fingerprinting. *Phys Rev Lett* 2001;87:167902. 10.1103/PhysRevLett.87.167902 11690244

[36] Mezzadri F . How to Generate Random Matrices From the Classical Compact Groups. *Notices of the American Mathematical Society* 2007;54:592–604.

[37] Kingma DP, Ba J. Adam: a method for stochastic optimization. arXiv preprint arXiv:1412.6980, 2014.

[38] Schuld M, Bergholm V, Gogolin C et al. Evaluating analytic gradients on quantum hardware. *Phys Rev A* 2019;99:032331. 10.1103/PhysRevA.99.032331

[39] Freund Y, Schapire RE. A desicion-theoretic generalization of on-line learning and an application to boosting. In Paul Vitanyi, ed. European Conference on Computational Learning Theory, Barcelona, Spain: Springer, 904:23–37, 1995, 10.1007/3-540-59119-2_166

[40] Mason L, Baxter J, Bartlett P et al. Boosting algorithms as gradient descent. *Adv Neural Inf Proces Syst* 1999;12:512–18.

[41] Friedman JH . Greedy function approximation: a gradient boosting machine. *Ann Stat* 2001;29:1189–232. 10.1214/aos/1013203451

[42] Breiman L . Random forests. *Mach Learn* 2001;45:5–32. 10.1023/A:1010933404324

[43] Grinsztajn L, Oyallon E, Varoquaux G. Why do tree-based models still outperform deep learning on typical tabular data? *Adv Neural Inf Proces Syst* 2022;35:507–20.

[44] Love MI, Huber W, Anders S. Moderated estimation of fold change and dispersion for RNA-seq data with DESeq2. *Genome Biol* 2014;15:1–21. 10.1186/s13059-014-0550-8 PMC4302049 25516281

[45] Litchfield K, Reading JL, Puttick C et al. Meta-analysis of tumor-and T cell-intrinsic mechanisms of sensitization to checkpoint inhibition. *Cell* 2021;184:596–614.e14. 10.1016/j.cell.2021.01.002 33508232 PMC7933824

[46] Zeng Z, Zhang T, Zhang J et al. A minimal gene set characterizes TIL specific for diverse tumor antigens across different cancer types. *Nat Commun* 2025;16:1070. 10.1038/s41467-024-55059-3 39900903 PMC11791090

[47] McInnes L, Healy J, Melville J. UMAP: uniform manifold approximation and projection for dimension reduction. arXiv preprint arXiv:1802.03426, 2018.

[48] Pérez-Salinas A, Cervera-Lierta A, Gil-Fuster E et al. Data re-uploading for a universal quantum classifier. *Quantum* 2020;4:226. 10.22331/q-2020-02-06-226

[49] Tucci RR . An introduction to cartan’s KAK decomposition for QC programmers. arXiv preprint quant-ph/0507171, 2005.

[50] Piveteau C, Sutter D. Circuit knitting with classical communication. *IEEE Trans Inf Theory* 2024;70:2734–45. 10.1109/TIT.2023.3310797

[51] Wierichs D, Izaac J, Wang C et al. General parameter-shift rules for quantum gradients. *Quantum* 2022;6:677. 10.22331/q-2022-03-30-677

