# Supplementary material for: Quantum ensembling methods for healthcare and life science
Source: Brief Bioinform. 2026 Jun 4;27(3):bbag280. doi: 10.1093/bib/bbag280 (PMC13236107; doi:10.1093/bib/bbag280)
Supplement: Supplementary_Material_Bbag280 [file supplementary_material_bbag280.zip › Briefings___Quantum_Ensemble_Supp_bbag280.pdf]

Supplementary Material: Quantum Ensembling methods for Healthcare and Life Science

Rhrissorrakrai et al.

May 11, 2026

Pseudo-code for variational training workflow

In the following section we provide pseudocode for the variational training workflows (i.e. soft-voting, bagging, and boosting). The **ForwardPass**$(x, \theta; n_s)$ of each learner encodes a classical feature (x) using amplitude encoding, executes the parameterized circuit $(\mathcal{U}(\theta)^{\ell})$, and assigns a label prediction y' using the state amplitudes of qubit [i] observed from n_s shots. To simplify our presentation, we omit the parameter n_s from **ForwardPass** as it is kept constant through the workflow, and distinguish the unique parameters of the ℓ-th learner using the superscript θ^{ℓ}. The gradient update step uses the analytic gradient formula [1]. To compute the gradient in parameter space two forward passes are required: $\theta_{i\pm}$ are the parameter vectors with the i-th element shifted by $\pm\pi$.

Algorithm 1 Supervised training a soft-voting ensemble of ℓ learners on n features, for T_{MAX} steps using learning rate α, and batch size b

procedure SoftVotingEnsemble$(\ell, n, T_{MAX}, \alpha, b)$
    function ForwardPass$((x, \theta))$
        $y'(x,\theta) \leftarrow \max\left[|\langle 0|\mathcal{U}(\theta)|\psi(x)\rangle|^2, |\langle 1|\mathcal{U}(\theta)|\psi(x)\rangle|^2\right]$
    end function
    function LossGradient$(\nabla_{y'}\mathcal{L})$
        $\nabla_{y'}\mathcal{L} \leftarrow \frac{\partial \mathcal{L}(y,y')}{\partial y'}$
    end function
    function ParameterGradient$(\nabla_{\theta} y'(x,\theta))$
        $\frac{\partial y'}{\partial \theta_i} \leftarrow \frac{1}{2}\big(\mathbf{ForwardPass}(\mathrm{x}, \theta_{\mathrm{i}+}) - \mathbf{ForwardPass}(\mathrm{x}, \theta_{\mathrm{i}-})\big)$
    end function
    $\mathcal{X} \leftarrow \mathcal{S}[i]$ ▷ Load training features
    $y \leftarrow \mathcal{Y}[i]$ ▷ Load training labels
    while $t \leq T_{MAX}$ **do**
        $\mathcal{B} \leftarrow (x_i, y_i) \sim \mathcal{X} \times \mathcal{Y}$ ▷ Draw batch of b samples
        $\mathbf{\Theta}(t) \leftarrow \bigcup_{\ell} \theta^{\ell}(t)$
        for all (x_i, y_i) in $\mathcal{B}$ **do**
            $y'_i \leftarrow \frac{1}{\ell}\sum_{\ell} \mathbf{ForwardPass}(x_i, \theta^{\ell})$
            $\mathbf{\Theta}(t+1) \leftarrow \mathbf{\Theta}(t) - \alpha\nabla_{y'_i}[\mathcal{L}(y_i, y'_i)]\nabla_{\theta}[y'_i(x,\theta)]$
        end for
    end while
end procedure

Algorithm 2 Supervised training a bagged ensemble of ℓ learners on n features, for T_{MAX} steps using learning rate α, and batch size b

procedure BaggedEnsemble($\ell, n, T_{MAX}, \alpha, b$)
    function ForwardPass((x, θ))
        $y'(x,\theta) \leftarrow \max\left[|\langle 0|\mathcal{U}(\theta)|\psi(x)\rangle|^2, |\langle 1|\mathcal{U}(\theta)|\psi(x)\rangle|^2\right]$
    end function
    function LossGradient($\nabla_{y'}\mathcal{L}$)
        $\nabla_{y'}\mathcal{L} \leftarrow \frac{\partial \mathcal{L}(y,y')}{\partial y'}$
    end function
    function ParameterGradient($\nabla_\theta y'(x,\theta)$)
        $\frac{\partial y'}{\partial \theta_i} \leftarrow \frac{1}{2}\big(\textbf{ForwardPass}(\mathrm{x}, \theta_{\mathrm{i}+}) - \textbf{ForwardPass}(\mathrm{x}, \theta_{\mathrm{i}-})\big)$
    end function
    $\mathcal{X} \leftarrow \mathcal{S} = \{S_1, S_2, \ldots, S_\ell\}$ ▷ Load training features and partition into ℓ subsets
    $y \leftarrow \mathcal{Y} = \{y_1, y_2, \ldots, y_\ell\}$ ▷ Load training labels and partition into ℓ subsets
    for i in $1 \ldots \ell$ **do** ▷ Train i-th learner on i-th subset
        while $t \leq T_{MAX}$ **do**
            $\mathcal{B} \leftarrow (x_j, y_j) \sim \mathcal{X}_i \times \mathcal{Y}_i$ ▷ Draw batch of b samples
            for all (x_k, y_k) in $\mathcal{B}$ **do**
                $y'_k \leftarrow \textbf{ForwardPass}(x_k, \theta^i)$
                $\theta^i(t+1) \leftarrow \theta^i(t) - \alpha \nabla_{y'_i}[\mathcal{L}(y_i, y'_i)] \nabla_{\theta^i}[y'_i(x, \theta^i)]$
            end for
        end while
    end for
end procedure

Algorithm 3 Boosted supervised training an ensemble of ℓ learners on n features, for T_{MAX} steps using learning rate α, and batch size b

procedure BoostedEnsemble($\ell, n, T_{MAX}, \alpha, b$)
  function ForwardPass((x, θ))
    $y'(x, \theta) \leftarrow \max \left[|\langle 0|\mathcal{U}(\theta)|\psi(x)\rangle|^2, |\langle 1|\mathcal{U}(\theta)|\psi(x)\rangle|^2 \right]$
  end function
  function LossGradient($\nabla_{y'}\mathcal{L}$)
    $\nabla_{y'}\mathcal{L} \leftarrow \frac{\partial \mathcal{L}(y, y'; w)}{\partial y'}$
  end function
  function ParameterGradient($\nabla_\theta y'(x, \theta)$)
    $\frac{\partial y'}{\partial \theta_i} \leftarrow \frac{1}{2}\big(\textbf{ForwardPass}(\mathrm{x}, \theta_{\mathrm{i}+}) - \textbf{ForwardPass}(\mathrm{x}, \theta_{\mathrm{i}-})\big)$
  end function
  $\mathcal{X} \leftarrow \mathcal{S} = \{S_1, S_2, \ldots, S_\ell\}$ ▷ Load training features, partition into subsets
  $\mathcal{W}(t = 0) \leftarrow 1/|\mathcal{X}|$ ▷ Assign equal importance weight
  $y \leftarrow \mathcal{Y} = \{y_1, y_2, \ldots, y_\ell\}$ ▷ Load training labels, partition into ℓ subsets
  for i in $1 \ldots \ell$ **do** ▷ Train i-th learner on i-th subset
    while $t \leq T_{MAX}$ **do**
      $\mathcal{B} \leftarrow (x_j, y_j, w_j) \sim \mathcal{X}_i \times \mathcal{Y}_i \times \mathcal{W}_i$ ▷ Draw batch of b samples
      for all (x_k, y_k, w_k) in $\mathcal{B}$ **do**
        $y'_k \leftarrow$ **ForwardPass**(x_k, θ^i)
        $\theta^i(t+1) \leftarrow \theta^i(t) - \alpha \nabla_{y'_i}[\mathcal{L}(y_i, y'_i; w_i)] \nabla_{\theta^i}[y'_i(x, \theta^i)]$
        $\epsilon[t] \leftarrow \Pr[y'_i \neq y_i], i \in \mathcal{X}$
        $\alpha_t \leftarrow \frac{1}{2} \ln \frac{1 - \epsilon[t]}{\epsilon[t]}$
        for w_i in $\mathcal{W}$ **do** ▷ update weights for all samples
          if $y'_i = y_i$ **then**
            $w_i \leftarrow w_i e^{-\alpha(t)}$
          else
            $w_i \leftarrow w_i e^{\alpha(t)}$
          end if
        end for
      end for
    end while
  end for
end procedure

References

[1] Maria Schuld, Ville Bergholm, Christian Gogolin, Josh Izaac, and Nathan Killoran. Evaluating analytic gradients on quantum hardware. *Physical Review A*, 99(3):032331, 2019.
